# The risk of anaphylaxis behind authorized COVID-19 vaccines: a meta-analysis

**DOI:** 10.1186/s12948-022-00167-y

**Published:** 2022-01-17

**Authors:** Marharyta Sobczak, Rafał Pawliczak

**Affiliations:** Department of Immunopathology, Faculty of Medicine, Division of Biomedical Science, Medical University of Lodz, St. Zeligowskiego 7/9, 90-752 Lodz, Poland

**Keywords:** COVID-19, Vaccines, Anaphylaxis, Coronavirus infection, SARS-CoV-2, Meta-analysis

## Abstract

**Background:**

A serious allergic reaction that may occur in response to medical products is anaphylaxis, which potentially can lead to anaphylactic shock. In the light of recent COVID-19 pandemic, much public attention had been paid to the severe allergic reactions occurring after COVID-19 vaccination. Therefore, in our study we would like to investigate the risk of authorized COVID-19 vaccines to induce anaphylactic reaction, anaphylactoid reaction, anaphylactic shock and anaphylactoid shock.

**Methods:**

We searched databases, such as PubMed, Web of Science and Embase and found eight articles about the incidence of anaphylactic and anaphylactoid reactions. Also, we used data from four databases from Canada, the U.S., the European Union and the United Kingdom. To calculate effect sizes, we used random effects model with inverse variance method. The risk ratio with 95% confidence interval were used for dichotomous outcomes. Statistical analysis was prepared in R. Results were considered statistically significant at p < 0.05.

**Results:**

The most cases of anaphylactic reaction, anaphylactoid reaction, anaphylactic shock and anaphylactoid shock were reported in female aged 18–85 years after BNT162b2 vaccine according to data from the EU. Analyzed COVID-19 vaccines can cause the anaphylaxis/anaphylactic reaction with risk of 106.99 (95% CI [39.95; 286.57], p < 0.0001, I^2^ = 59%), whereas the anaphylactoid reaction, anaphylactic and anaphylactoid shocks with risk of 113.3 (95% CI [28.11; 456.53], p < 0.0001), 344.2 (95% CI [85.77; 1381.39], p < 0.0001), 14.9, 95% CI [1.96; 112.79], p = 0.009), respectively.

**Conclusions:**

Our meta-analysis shows that the risk of anaphylactic reaction, anaphylactoid reaction, anaphylactic shock and anaphylactoid shock do not occur only after mRNA COVID-19 vaccines. Therefore, vaccination centers should be prepared to render assistance in the event of a reaction in all cases.

## Introduction

The first identified case of COVID-19 occurred in Wuhan in December 2019. Spreading around the world and infected 114 countries in March 2020, COVID-19 was classified as a pandemic [1]. According to WHO COVID-19 dashboard [2], around 245.37 million cases of COVID-19 infections have been reported on October 29, 2021 with around 4.98 million deaths. An important part of fighting a pandemic is finding an effective yet safe vaccine. There are different types of COVID-19 vaccines in development stages. On October 29, 2021 around 6.84 billion doses of COVID-19 vaccines have been distributed over the world [2]; whereas on October 30, 2021 there were 1053 clinical studies of COVID-19 vaccines [3].

In the European Union (EU), there are four COVID-19 vaccines authorized for use: BNT162b2, mRNA-1273, ChAdOx1 nCoV-19 and Ad26.COV2.S [4]. BNT162b2 and mRNA-1273 are the lipid nanoparticle-formulated mRNA vaccines that encoding SARS-CoV-2 spike glycoprotein with proline mutation in two sites [5–7]. These vaccines were authorized for use in human subjects by U.S. FDA (Food and Drug Administration) in December 2020: December 11, 2020 for BNT162b2 vaccine and December 18, 2020 for mRNA-1273 vaccine, respectively [8]. In contrast, the authorization approval of these vaccines in the European Union came a little later: December 21, 2020 for BNT162b2 vaccine and January 6, 2021 for mRNA-1273 vaccine [4]. The results of phase 2/3 of randomized clinical trial of BNT162b2 vaccine showed that this vaccine has 95% efficiency against COVID-19 after full vaccination with two doses in participants older than 16 years [7]. Similar results showed the phase 3 randomized clinical trial of mRNA-1273 vaccine—94.1% efficiency in participants older than 18 years [9]. In addition, the safety and efficiency of BNT162b2 vaccine were studied on adolescents at the age of 12 to 15 years and demonstrated 100% vaccine efficacy [5]. Therefore, the BNT162b2 vaccine is currently recommended for use in adolescents aged 12–15 years according to U.S. FDA emergency use authorization from May 10, 2021 [8]. In contrast, the other two vaccines (ChAdOx1 nCoV-19 and Ad26.COV2.S) have been authorized in the EU on January 29, 2021 and March 11, 2021, respectively [4]. These vaccines include a viral vector that is unable to replicate and encodes SARS-CoV-2 spike glycoprotein. ChAdOx1 nCoV-19 vaccine contains chimpanzee adenovirus vector, while Ad26.COV2.S contains human adenovirus serotype 26 [10–13]. As of February 27, 2021 Ad26.COV2.S vaccine has been approved by the U.S. FDA [8]. The randomized clinical trial in phase 3 showed the efficiency of this single dose vaccine at level 67% after 14 days of vaccination and 66% after 28 days of vaccination [13]. An interim analysis of four ChAdOx1 nCoV-19 vaccine randomized clinical trials showed the vaccine efficiency equals 70.4% after full vaccination with two doses [14]. In addition, an exploratory analysis of randomized clinical trial in phase 2/3 showed the efficiency of vaccine was 81.5% against non-B.1.1.7 variants of COVID-19 and 70.4% against B.1.1.7 variant [15].

An important aspect of the new vaccines is their safety. While in usual situation it takes more time to produce new vaccines, the process of developing and producing COVID-19 vaccines was accelerated due to urgent need to fight the pandemic [16]. Potentially all vaccines can cause anaphylaxis [17]. Anaphylaxis is severe, systemic, immediate allergic reaction. There are different types of anaphylaxis. The first one is uniphasic, which occurs the most commonly and very quickly—within 30–60 min. The second one is biphasic, which recurs after the first symptoms have disappeared without re-exposure to the trigger. And the last one is persistent, which can last several days or even weeks [18]. In 2004, Brown [19] developed new system grading of anaphylaxis, defining the severe anaphylaxis as failure of respiratory or cardiovascular system. This grading consists of three levels: mild pertaining to skin and subcutaneous tissue, moderate concerns the gastrointestinal, cardiovascular or respiratory systems, and severe, which includes neurologic compromise, hypoxia or hypertension. Therefore, assessment of the risk of anaphylaxis after vaccination is an crucial point in vaccine safety research [17]. The typical symptoms of anaphylaxis are urticaria or angioedema, bronchospasm and hypotension [20]. During anaphylaxis after exposure to allergen, mast cells or basophils produce inflammatory immune mediators caused by IgE (immunoglobulin E) binding to high affinity receptors FcεRI leading to crosslinking of receptors and activation of these cells. Produced mediators, such as histamine, proteases and leukotrienes, prostaglandins can lead to bronchial smooth muscle contraction, vasodilation, as well as increased mucus production and vascular permeability [20–22]. Besides anaphylactic reaction, there is anaphylactoid reaction that has similar symptoms and treatment, but other mechanisms consisting of the complement activation or activation of bradykinin cascade and mast cells or basophils direct activation [20, 22]. Therefore, in our study, we would like to compare COVID-19, up-to-date registered, vaccines in terms of incidence of anaphylactic reaction, anaphylactoid reaction, anaphylactic shock, and anaphylactoid shock after vaccine administration. Our study showed that anaphylaxis may occur after administration of either mRNA- or virus-based vaccine. Interestingly, the frequency of anaphylaxis among the vaccinated group is higher in female patients which may suggest potential involvement of hormonal regulation in origin of anaphylaxis.

## Methods

### Search strategy, data search and extraction

For this meta-analysis, databases, such as Embase, PubMed and Web of Science were searched to find literature published before October 14, 2021 using the following search strategy: *((((COVID-19) OR (coronavirus infection)) OR (SARS-CoV-2)) AND ((vaccine) OR (vaccination))) AND (((((anaphylaxis) OR (anaphylactic reaction)) OR (anaphylactic shock)) OR (anaphylactoid reaction)) OR (anaphylactoid shock))*. We included all reports and studies published in English about anaphylaxis or anaphylactoid incidents caused by authorized COVID-19 vaccines, such as BNT162b2, mRNA-1273, ChAdOx1 nCoV-19 and Ad26.COV2.S. Additionally, we used data from *EudraVigilance–European database of suspected adverse drug reaction reports* [23] about the number of reported cases of anaphylactic reaction, anaphylactoid reaction, anaphylactic shock, and anaphylactoid shock after COVID-19 vaccination with vaccines authorized in the European Union: BNT162b2, mRNA-1273, ChAdOx1 nCoV-19 and Ad26.COV2.S, up to October 9, 2021. Information about the number of doses of the foregoing COVID-19 vaccines administered to EU/EEA countries downloaded from COVID-19 Vaccine Tracker from *European Centre for Disease Prevention and Control* [24] as of 41 week of 2021 year (October 10, 2021). Also, we used data from *CDC—Centers for Disease Control and Prevention* [25] (up to October 8, 2021), *Public Health Agency of Canada* [26] (up to October 1, 2021) and *Medicines and Healthcare products Regulatory Agency* [27] (up to October 6, 2021). Literature search was prepared according to PRISMA flow diagram [28].

### Statistical analysis

The statistical analysis was prepared using R version 4.0.5. Additionally, we used GraphPad Prism 8.0.2 for graph preparation. To compare the cases of anaphylaxis caused by COVID-19 vaccines with absent of vaccination, the risk ratio (RR) with 95% confidence interval (CI) were used for dichotomous outcomes. We used data about number of anaphylactic reactions, anaphylactoid reactions, anaphylactic shocks, and anaphylactoid shocks per administrated doses of particular COVID-19 vaccines. If such data were not available, we used the rate of anaphylactic reactions, anaphylactoid reactions, anaphylactic shocks, and anaphylactoid shocks per million administrated doses of particular COVID-19 vaccines. We assumed that the control group would be unvaccinated individuals with an anaphylactic reaction, anaphylactoid reaction, anaphylactic shock, and anaphylactoid, which prompted by COVID-19 vaccines at rate of 0. Random effects model using inverse variance method was used to calculate effect sizes. I^2^ statistics was used to evaluate the heterogeneity of studies: I^2^ < 40% may not be important; 30% < I^2^ < 60% means moderate heterogeneity; 50% < I^2^ < 90% means substantial heterogeneity; I^2^ > 75% means considerable heterogeneity [29]. Results of this meta-analysis were considered statistically significant at p < 0.05.

## Results

**Fig. 1:**
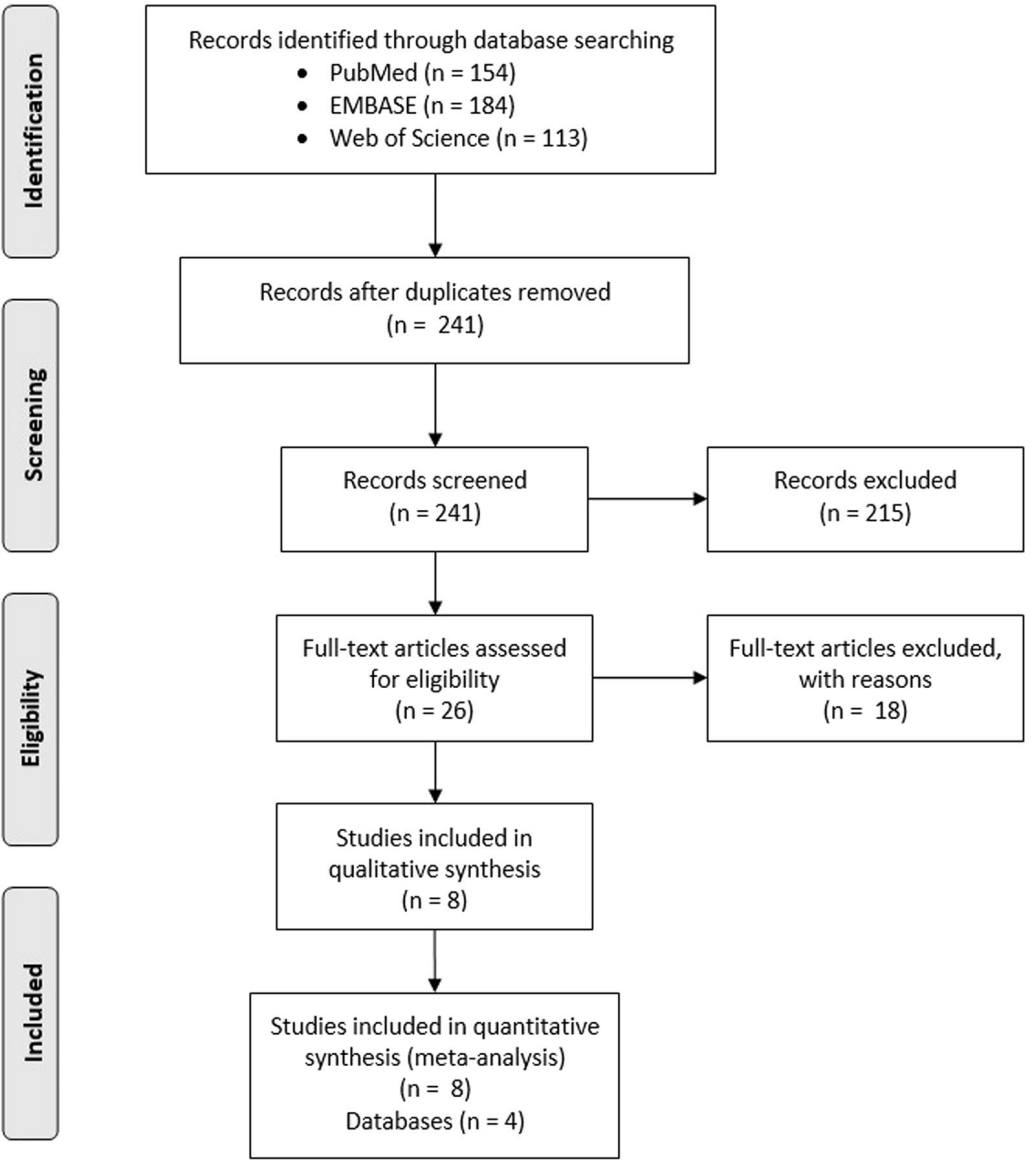
Selection of studies for meta-analysis

After electronic searching, 241 non-duplicated records were identified as shown on Fig. 1. Next, we excluded 215 records after titles and abstracts screening and 18 after full-text screening. Finally, our meta-analysis contains 2 research letters from Japan [30] and U.S. [31], an interim analysis from 8 data-contributing health plans in the U.S. [32], and 5 reports from U.S. [33–35], South Korea [36] and Japan [37]. Additionally, we used data from 4 databases that contain information about anaphylaxis and anaphylactoid reaction incidence after COVID-19 vaccination of population in the European Union [23], U.S. [25], Canada [26] and United Kingdom [27].

### Gender association with anaphylaxis after COVID-19 vaccines

**Fig. 2:**
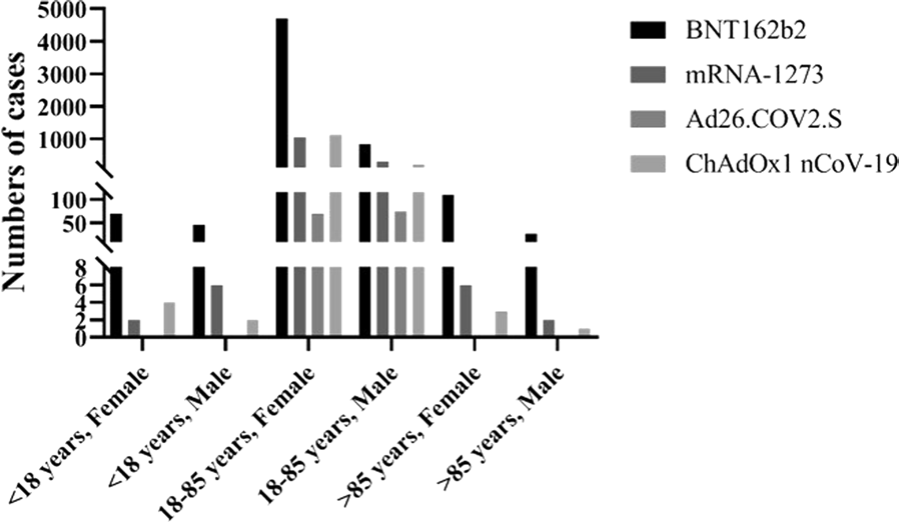
The number of all anaphylactic and anaphylactoid reactions and shocks after COVID-19 vaccines. Data included cases from the European Union before October 9, 2021 based on *EudraVigilance–European database of suspected adverse drug reaction reports* [23]

Based on data from *EudraVigilance–European database of suspected adverse drug reaction reports* [23], we indicated that anaphylactic reactions, anaphylactoid reactions, anaphylactic shock, and anaphylactoid shock may occur after all approved COVID-19 vaccines. Of these, most cases of these reactions were reported in female aged 18–85 years after vaccination with BNT162b2 vaccine, as shown on Fig. 2.

### Anaphylaxis or anaphylactic reactions after COVID-19 vaccines

**Fig. 3:**
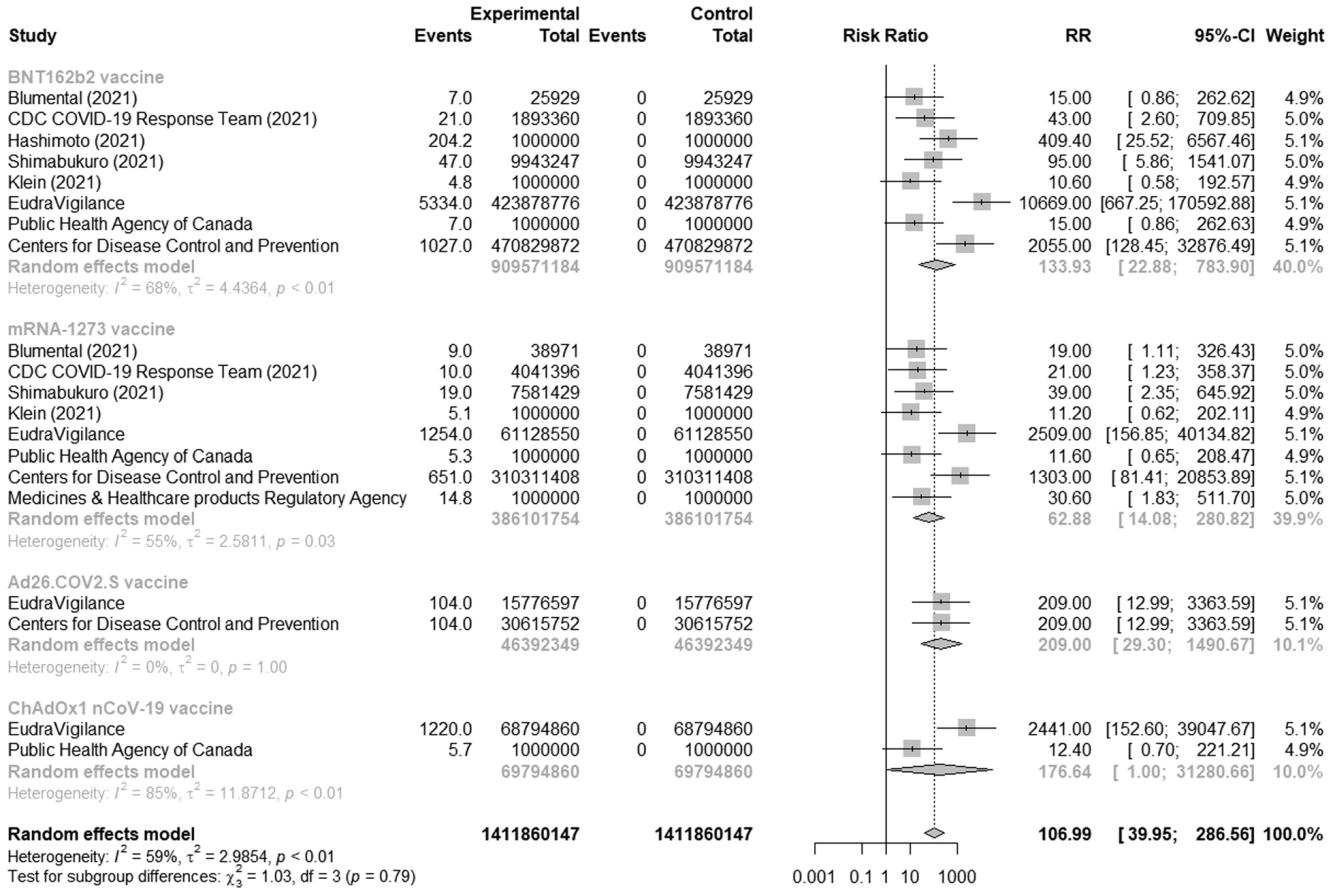
Risk of anaphylaxis/anaphylactic reactions after COVID-19 vaccines compared to unvaccinated people

In this analysis, we used 6 articles [30–35] and 4 databases [23, 25–27] that indicates the number of anaphylaxis or anaphylactic reactions after COVID-19 vaccination. Because of high heterogeneity, we performed the subgroup analysis and found that the overall risk of anaphylaxis or anaphylactic reaction after vaccination with authorized COVID-19 vaccines was 106.99 (95% CI [39.95; 286.57], p < 0.0001, I^2^ = 59%) with substantial heterogeneity, as shown on Fig. 3. Moreover, the highest risk of anaphylaxis or anaphylactic incidences was after adenovirus-vector vaccine Ad26.COV2.S and equals 209 (95% CI [29.3; 1490.67], I^2^ = 0%) without heterogeneity, while the lowest risk was 62.89 (95% CI [14.08; 280.84], I^2^ = 55%) with substantial heterogeneity after mRNA vaccine mRNA-1273.

### Anaphylaxis including anaphylactoid reactions after COVID-19 vaccines

**Fig. 4:**
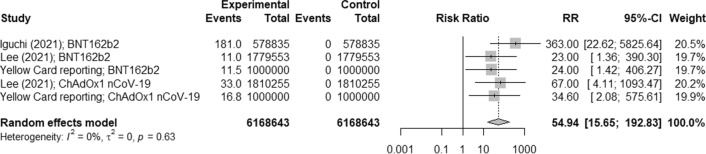
Risk of anaphylaxis including anaphylactoid reaction after COVID-19 vaccines compared to unvaccinated people

A few reports [36, 37] and database [27] reported the cases of anaphylaxis and anaphylactoid reactions after BNT162b and ChAdOx1 nCoV-19 vaccines. As shown Fig. 4, the risk of anaphylaxis and anaphylactoid reactions caused by these vaccines was 54.94 (95% CI [15.65; 192.83], p < 0.0001, I^2^ = 0%).

### Anaphylactic shock, anaphylactoid reaction and anaphylactoid shock after COVID-19 vaccines

**Fig. 5:**
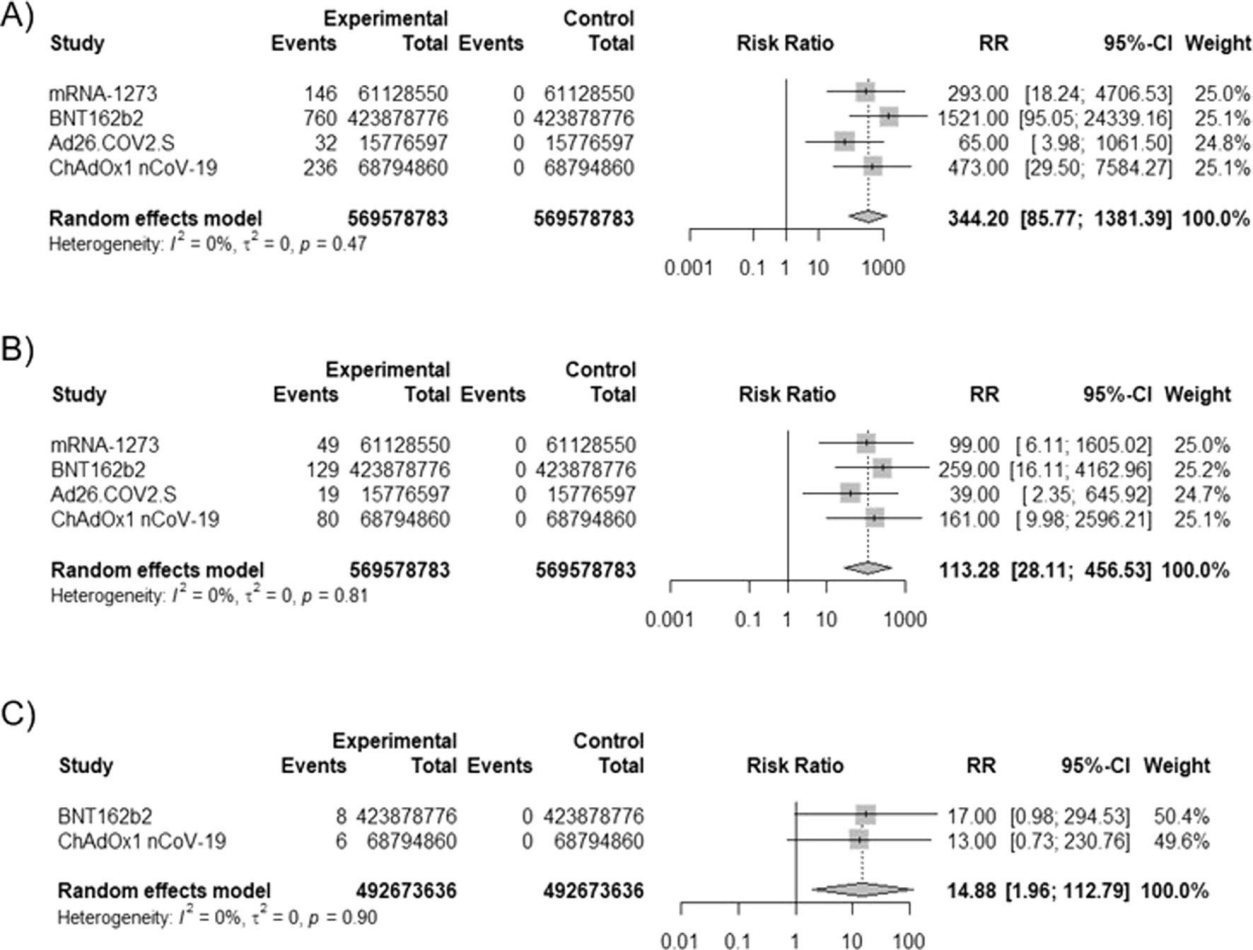
Risk of anaphylactic shock, anaphylactoid reaction, anaphylactoid shock after COVID-19 vaccines. **A** risk of anaphylactic shock after vaccination, **B** risk of anaphylactoid reaction after vaccination, **C** risk of anaphylactoid shock after vaccination; based on *EudraVigilance–European database of suspected adverse drug reaction reports* [23]

Additionally, we analyzed the incidents of anaphylactic shock, anaphylactoid reaction and anaphylactoid shock after COVID-19 vaccines authorized in the EU based on data from *EudraVigilance–European database of suspected adverse drug reaction reports* [23]*.* COVID-19 vaccines caused more anaphylactic shock (RR = 344.2, 95% CI [85.77; 1381.39], p < 0.0001), anaphylactoid reaction (RR = 113.3, 95% CI [28.11; 456.53], p < 0.0001) and anaphylactoid shock (RR = 14.9, 95% CI [1.96; 112.79], p = 0.009) without heterogeneity (Fig. 5).

## Discussion

Our meta-analysis shows that after vaccination with COVID-19 vaccines, there is a risk of incidence of anaphylactic reaction, anaphylactoid reaction, anaphylactic shock, and anaphylactoid shock. In the EU, the most numbers of these reactions were reported for vaccinated people at age between 18 and 85 years, and the least numbers in vaccinated cohort at age less than 18 years and above than 85 years. Moreover, the most numbers of anaphylaxis was reported in vaccinated females compared to males at age between 18 and 85 years. Interestingly, in this cohort of females, the most numbers of reactions were reported after administration of BNT162b2 vaccine, which can be explained by the predominant number of administrated doses of this vaccine compared to other vaccines [24]. It can suggest that gender plays an important role in anaphylaxis. In general, studies showed that females have more predisposition to drug allergy, for example to penicillin. However, this association has not been observed in children. This can be explained by sex hormones. Estrogen can enhance activity of endothelial nitric oxide synthase, which was observed in study carried out on mice. This led to elevation of the vascular permeability as well as the severity of anaphylaxis [38, 39]. On the other hand, progesterone can inhibit production of histamine from mast cells [39]. Similar results in the subject of predominant of female cases of anaphylaxis in adults were observed in study, in which rates of anaphylaxis after vaccination in period between January 1, 2009 and December 31, 2011 were analyzed [17].

Among analyzed vaccines, Ad26.COV2.S vaccine had the highest risk of anaphylaxis/ anaphylactic reaction in the U.S. and EU. Moreover, in the EU, cases of anaphylactic shock and anaphylactoid reaction were reported, but the risk of these was the lowest among analyzed vaccines. Other virus vector vaccine ChAdOx1 nCoV-19 also had risk of anaphylaxis/ anaphylactic reaction higher than mRNA vaccines. This vaccine also can cause the anaphylactic shock, anaphylactoid reaction and anaphylactoid shock. On the other hand, data from Korea concerning adverse events after vaccination of 998 healthcare workers showed that there was no cases of anaphylaxis after first dose of the ChAdOx1 nCoV-19 vaccine [40]. Among mRNA vaccines, the higher risk of anaphylaxis or anaphylactic reaction, anaphylactoid reaction as well as anaphylactic and anaphylactoid shocks occurred after BNT162b2 vaccine administration, especially in the EU. However, a report from Ontario in period between December 13, 2020 and March 6, 2021 noted anaphylaxis cases the rates of 32.7 and 38.0 per million doses administered of mRNA-1273 and BNT162b2, respectively [41]. Among health care providers from Baylor Scott and White Health from Texas, which were vaccinated with first dose, only 3 persons had anaphylaxis what corresponds to 0.01% of vaccinated medical staff members [42]. In overall, in U.S. after first month of vaccination in period between December 14, 2020 and January 13, 2021, 4.5 per million cases of anaphylaxis after doses administered of both mRNA vaccines have been reported [43].

Considering potential causes of anaphylaxis reactions after COVID-19 vaccine administration, one can think of the vaccine component, such as polyethylene glycol (PEG2000), which is contained in mRNA vaccines—BNT162b2 and mRNA-1273, while viral vector vaccines (ChAdOx1 nCoV-19 and Ad26.COV2.S) contain polyethylene glycol derivatives, called polysorbate 80 [44, 45]. Polyethylene glycols are group of polymers, which are formed from ethylene oxide during the polymerization reaction. These polyether compounds are used in different industry, such as cosmetics, medicine, household products and food. The pegylation of drugs may increase the circulation time, because of drug protection from immune degradation as well as metabolism. In case of mRNA COVID-19 vaccines, pegylated nanoparticles can protect mRNA against enzymatic degradation increasing its stability [45, 46]. Moreover, mRNA may bind to PAMP (pathogen-associated molecular pattern) receptors as well as activate contact system protein, which in turn, can lead to anaphylactoid reactions. This is another reason, why mRNA COVID-19 vaccines are encapsulated into nanoparticles [39]. The mechanism of hypersensitivity reactions against PEG is not fully understood. Studies showed that IgM and IgG antibodies can form against PEG in humans, which for the first time was reported in 2005 in trials of Pegloticase. Similar effect was observed after administration of PEG asparaginase used in chemotherapy [39]. These formed IgG and IgM antibodies can activate complement as well as release the mediators, which is called complement activation-related pseudoallergy (CAPRA). In addition to the above, PEG may induce the formation of IgE antibodies [46]. In Singapore in April 2021, there were 20 cases of anaphylaxis after 2 213 888 doses of BNT162b2 and mRNA-1273 vaccines. Among these cases, Lim XR et al. [47] analyzed anti-BNT162b2 and anti-PEG antibodies for 3 patients. They detected IgG and IgM antibodies against BNT162b2 vaccine in all samples, as well as the higher level of IgG and IgM against PEG in 2 samples. However, IgE antibodies against BNT162b2 vaccine were not detected, from which it can be concluded that these cases of anaphylaxis were examples of CAPRA, not of typical allergic reaction. Due to the small number of studied patients, it cannot be concluded that this mechanism alone is responsible for the development of anaphylaxis after BNT162b2 vaccine.

In contrast to PEG, polysorbates are components of many used vaccines and medicines including monoclonal antibodies as well as biological agents. Because these PEG derivatives have less molecular weight than PEG, they are less likely to cause allergic reactions. Although, there are reports of anaphylaxis caused by polysorbates in animal models via IgE-independent mechanism, but not much cases were reported in humans [45]. Interesting case of biphasic anaphylaxis was reported after vaccination with first dose of BNT162b2 vaccine. After skin and intradermal testing, the patient was found to be negative for PEG, but different results for drugs containing polysorbate 80, which is not present in the BNT162b2 vaccine: skin test for triamcinolone acetonide was negative, but intradermal test was positive, then test for Prevnar-13 was negative, but for Refresh sterile eye drops was positive. Additionally, the test for BNT162b2 vaccine was also negative. The authors suggested that the particular case of anaphylaxis arose from a cross-reaction of PEG and polysorbate 80, however negative results of skin tests for PEG as well as methylprednisolone do not support this suggestion. The reason for such discrepancies may be the low concentration of PEG needed to bring on positive test reaction [48].

The crucial point in anaphylaxis reaction after COVID-19 vaccination is correct and quick management after vaccine administration. According to *Centers for Disease Control and Prevention* [25] recommendations on anaphylaxis management after COVID-19 vaccines, at the vaccination sites the qualified healthcare personnel who could identify the symptoms and administer the epinephrine should be available. Moreover, recommended observation time after vaccination equals 15 min, but it is extended to 30 min for people with a history of anaphylaxis induced by any cause or an immediate allergic reaction to other vaccine or injectable therapy as well as for people with a contraindication to other types of COVID-19 vaccines. If anaphylaxis is suspected, breathing, airway, circulation and mental activity of patient should be assessed and emergency medical services should be immediately contacted. Next, the patient should be place in a supine position. The first-line drug, epinephrine, should be administered intramuscularly in dose 0.3 mg with maximum dose 0.5 mg every 5–15 min.

In summary, our meta-analysis showed that all authorized COVID-19 vaccines may cause anaphylactic reaction, anaphylactoid reaction, anaphylactic shock, and anaphylactoid shock. However, our study has several limitations. First, in the study, we used data from reports as well as databases, and updates of data in databases is not well-coordinated. Second, not all anaphylaxis cases may have been reported correctly and timely. All this may have resulted in a slight deviation of our calculations from reality. In addition, we included all reports in our analysis, not just those assessed by the Brighton criteria.

## Conclusion

Allergic reaction, especially anaphylaxis, may be a serious problem in COVID-19 vaccination process. Therefore, it is important to acknowledge the risk factors predisposing to the anaphylaxis caused by vaccination. Of note, in case of administration of any up-to-date registered COVID-19 vaccines, proper anaphylaxis precautions as well as adequately trained medical staff are required.

## Data Availability

All data generated or analyzed during this study are included in this published article.
